# COVID-19 Not Hypertension or Diabetes Increases the Risk of Preeclampsia among a High-Risk Population

**DOI:** 10.3390/ijerph192416631

**Published:** 2022-12-10

**Authors:** Rachael Morris, Ahmed S. Z. Moustafa, Wondwosen Kassahun-Yimer, Sarah Novotny, Brittney Billsby, Amira Abbas, Kedra Wallace

**Affiliations:** 1 Department of Obstetrics & Gynecology, University of Mississippi Medical Center, Jackson, MS 39216, USA; 2 Department of Data Sciences, University of Mississippi Medical Center, Jackson, MS 39216, USA; 3 School of Medicine, University of Mississippi Medical Center, Jackson, MS 39216, USA; 4 Department of Pharmacology & Toxicology, University of Mississippi Medical Center, Jackson, MS 39216, USA; 5 Myrlie Evers-Williams Institute for Elimination of Health Disparities, Jackson, MS 39213, USA

**Keywords:** diabetes, hypertension, obesity, preeclampsia, SARS-CoV2

## Abstract

Severe acute respiratory syndrome coronavirus 2 (SARS-CoV2) infection has been associated with greater morbidity and increased mortality in certain populations, such as those with chronic medical conditions, the elderly, and pregnant women. Our goal was to determine if COVID-19 infection during pregnancy increased the risk of preeclampsia in a population of women with increased risk factors for preeclampsia. We present a prospective observational matched case–control study of 100 deliveries with confirmed SARS-CoV2. Specifically, we investigated the maternal and neonatal outcomes in a high-risk population of pregnant women. Among women with COVID-19, the severity of symptoms was associated with the incidence of preeclampsia, but not with pre-existing diabetes or hypertension. Women with more severe symptoms were more likely to delivery pre-term with smaller babies. After adjusting for diabetes, hypertensive women with COVID-19 had an increased risk of preeclampsia aOR4.3 [1.5,12.4] compared to non-hypertensive women with COVID-19. After adjusting for hypertension, women with diabetes and COVID-19 had an increased risk of preeclampsia aOR3.9 [1.2,12.5]. This relationship was not seen among women without COVID-19. For women who had pre-existing diabetes or hypertension, the risk of developing preeclampsia was only increased if they were also diagnosed with COVID-19, suggesting that in our population of women the risk of preeclampsia is not associated with pre-existing diabetes or hypertension.

## 1. Introduction

Pregnant women with coronavirus disease 2019 (COVID-19), caused by the severe acute respiratory syndrome coronavirus 2 (SARS-CoV-2) are at higher risk of severe disease and adverse clinical outcomes compared to nonpregnant women with COVID-19 [1,2]. Surveillance data from the Centers for Disease Control and Prevention suggest that pregnancies complicated by COVID-19 are associated with an increased risk of hospitalization, ICU admission, and mechanical ventilation [3]. A report of pregnant women in the United States found that in comparison to women without COVID-19, those infected with COVID-19 during pregnancy had an increased risk of several adverse obstetrical outcomes [4]. It was found that women with COVID-19 had an increased risk of preterm delivery, stillbirth, preeclampsia (PreE) and placental abruption. Similar findings have also been reported from other studies, where authors have reported that COVID-19 during pregnancy increased the risk of preterm delivery, stillbirth, PreE and premature rupture of membranes [5,6]. 

From early in the COVID-19 pandemic it was recognized that certain factors increased the severity of COVID-19 infection. These risk factors included Black race or Hispanic ethnicity, obesity, and chronic comorbidities such as hypertension and/or diabetes [7]. These are among the same factors that have been associated with increased risk of preterm birth and PreE [8]. What is interesting about these studies evaluating obstetric outcomes in women with COVID-19 is that the data was collected from general populations and not high-risk populations [4,5]. The aim of the current study was to determine if COVID-19 increased the risk of PreE in a population of women at increased risk for PreE. 

## 2. Materials and Methods

This is a prospective observational matched case–control study conducted at the University of Mississippi Medical Center’s Winfred L. Wiser Hospital for Women & Infants located in the United States. Electronic medical records from women who were part of an approved Institutional Review Board COVID-19 Registry (IRB# 2020-0134) were selected for the current analysis. From 14 April–17 October 2020 100 women with COVID-19 or who had a diagnosis of COVID-19 during the current pregnancy were admitted for delivery. This period corresponds with the first 100 deliveries at our hospital during the COVID-19 pandemic. Per the CDC’s 2019 guidelines COVID-19 was diagnosed following positive detection of SARS-CoV-2 by nasopharyngeal swab and a quantitative polymerase-chain-reaction test. All cases were categorized as asymptomatic, mild, moderate, severe or critically ill based on current government guidelines and recommendations [9,10].

COVID-19 patients were matched to women without COVID-19 who delivered at our hospital from 18 January 2020–27 December 2020. Matches were based by maternal age ± 1-year, race/ethnicity, body mass index (BMI) class at the time of delivery, parity and presence of chronic hypertension or diabetes (Type I, Type II or gestational). 

### Statistical Methods

Continuous variables were presented as means ± standard deviations and categorical variables were described using frequencies and percentages. Associations between participant characteristics, severity of COVID-19 and/or intensive care unit (ICU) admission were tested using Kruskal–Wallis, Chi-square, Fisher’s exact test, T-test and one-way analysis of variance. Multivariable logistic regression models were used to assess associations between PreE, hypertension and diabetes. All statistical analyses were performed with SAS version 9.4 (SAS Institute Inc., Cary, NC, USA), with 2-sided *p* < 0.05 considered significant.

## 3. Results

We first examined the impact of COVID-19 to see if there were differences in delivery outcome based on race, comorbid risk factors and severity of COVID-19. Women were diagnosed with COVID-19 at 33.7 ± 5.7 weeks and delivered at 36.82 ± 3.7 weeks. Women who tested positive for COVID-19 in the 2nd trimester (17%) were significantly more likely to deliver prematurely relative to women testing positive in their 3rd trimester (35.02 ± 4.9 vs. 37.57 ± 2.4 weeks, *p* = 0.05). When severity of COVID-19 was assessed, 98/100 women were able to be classified. Data was not available for 2 women who did not receive COVID-19 care at our facility and we were unable to obtain medical records for review. From these 98 women, 50 were classified as asymptomatic and 3 as critically ill (Table 1). There was a statistical difference in the range of BMIs between classifications (*p* = 0.03, Table 1). Asymptomatic women had the lowest mean BMI and women who were critically ill had the highest mean BMI.

There were no statistical differences in racial/ethnic distribution, however all 6 of the non-Hispanic white women who had COVID-19 had the least severe of COVID-19 manifestations (Table 1). Severity of COVID-19 was not linked with hypertensive or diabetic status (*p* = 0.35). When the relationship between severity of COVID-19, hypertension/diabetic status and race was examined, there was no association (*p* = 0.26), suggesting that neither maternal race/ethnicity nor the presence of hypertension or diabetes contributed to severity of COVID-19. When pregnancy complications were evaluated, severity of COVID-19 was not associated (*p* = 0.35, Table 1). Gestational age at delivery decreased as COVID-19 severity increased (*p* = 0.02) and was accompanied by a statistical difference in the distribution of preterm birth with 50% of women with moderate to critical illness delivering preterm (*p* = 0.006, Table 1). 

**Figure 1:**
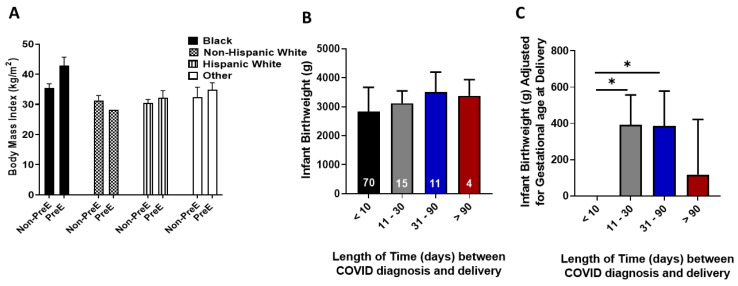
(**A**). Body mass index was compared between women with and without preeclampsia (PreE) within racial and ethnic groups. (**B**). Infant birthweight was compared for the length of time between gestational age at COVID-19 diagnosis and gestational age at delivery. The number in white denotes the number of women in each category. (**C**). When differences in infant birthweight was adjusted for gestational age at delivery and then compared to the time interval between gestational age at COVID-19 diagnosis and gestational age at delivery, infants who were born less than 10 following a positive COVID-19 diagnosis had statistically lower weights relative to infants born 11–90 days following a positive maternal COVID-19 diagnosis. * denotes *p* < 0.05 between the indicated groups.

There was a significant relationship between COVID-19 severity and incidence of PreE (*p* = 0.009, Table 1). Women classified with moderate–critical COVID-19 were more likely to develop PreE relative to women classified with asymptomatic–mild symptoms. We evaluated the relationship of hypertension and diabetes and found that 62.5% of women with COVID-19 and PreE also had hypertension or diabetes (*p* = 0.01). When we included maternal race and ethnicity (mother being Black or of Hispanic descent) into the analysis, 58.3% of women were Black or of Hispanic descent, had hypertension or diabetes and developed PreE (*p* = 0.006). Mean BMI for women with PreE increased compared to women without PreE in the same racial/ethnic category among all women except non-Hispanic white women (Figure 1A, *p* = 0.009).

Among the women with severe or critical illness, nine (69.2%) women required admission to the ICU. Compared to women with COVID-19 not needing ICU admission there were no statistical differences in maternal age (*p* = 0.89). There were no statistical differences between groups in gestational age at COVID-19 diagnosis (*p* = 0.26) or preterm delivery (*p* = 0.22, Table S1). ICU admissions were among women who were not non-Hispanic white (*p* = 0.03), and had a higher average BMI (39.76 ± 10.5 kg/m^2^ *p* = 0.05). Diagnosis of hypertension or diabetes did not increase the risk of ICU admission (*p* = 0.48). Women admitted to the ICU were more likely to deliver earlier than women not requiring ICU care (*p* = 0.008) and delivered babies at a lower birthweight (*p* = 0.008, Table S1). 

One patient in our study died due to complications from COVID-19 (diagnosed at 32.2 weeks). She was an obese (46.6 kg/m^2^) primigravida 23-year old black mother with chronic hypertension. She was admitted for acute hypoxic respiratory failure in the setting of COVID-19 and underwent emergent cesarean section shortly thereafter due to worsening respiratory status. There were no obstetrical complications (i.e., placental abruption, chorioamnionitis) with the delivery (33.0 weeks) and the patient was transferred to the ICU in stable condition. By post-partum day 16 the patient’s condition had declined to multiple organ failure which contributed to her death.

Infant birthweight decreased as maternal COVID-19 severity increased (*p* = 0.04). There were 7 infants who died following delivery, however this was not associated with COVID-19 severity (*p* = 0.09, Table 1). Table S2 provides details considered pertinent to the death of these infants. There was not a statistical difference in NICU admission (*p* = 0.13) or NICU length of stay (*p* = 0.14, Table 1) when COVID-19 severity was assessed.

The time interval between testing positive for COVID-19 and delivery was determined to assess the impact of COVID-19 on infant birthweight. There was no statistical difference in birthweight based on the time interval between COVID-19 diagnosed and delivery (*p* = 0.07; Figure 1B). Infant birthweight was adjusted by gestational age at delivery and infants born to women who had COVID-19 for 11–30 days (average weight difference 390.9 g, *p* = 0.02) or 31–90 days (average weight difference 385.95 g, *p* = 0.05) prior to delivery, weighed significantly more relative to those born to women who tested positive for COVID-19 within 10 days of delivery (Figure 1C). There was a difference in gestational age at delivery based on the time interval between COVID-19 diagnosis and delivery (*p* = 0.03) with delivery age increasing as the time interval increased.

We next compared our cases to controls to determine if the differences seen in our COVID-19 population were driven by COVID-19 or are more inherent to the high-risk population. The comparative analysis included 200 women undergoing a singleton pregnancy of whom 100 had confirmed COVID-19 infection (cases) and 100 women without COVID-19 (controls). Two women with COVID-19 were Native American and there were not any comparable control women in the same racial/ethnic category within the time frame, therefore two black women were chosen as matches. Among the women with in the chronic hypertension category, 2 of the cases had gestational hypertension and 5 of the controls had gestational hypertension as opposed to chronic hypertension. As indicated in Table 2, there was no statistical differences among any of the matched variables between groups. 

There was no statistical difference in the mean gestational age at delivery for women with COVID-19 (36.82 ± 3.7 wks) vs. controls (37.14 ± 3.1 wks, *p* = 0.51, Table 2). Neither was preterm delivery increased among women with COVID-19 (*p* = 0.43). Cesarean delivery was not increased in women with COVID-19 as 44% of these women had a cesarean delivery vs. 40% among controls (*p* = 0.57). There were no statistical differences among pregnancy complications between the two groups (*p* = 0.31), nor was there a difference in the incidence of PreE between groups (*p* = 0.74).

As both diabetes and hypertension are independent risk factors for COVID-19 and PreE, we adjusted for these variables. PreE among hypertensive women (i.e., women with a diagnosis at admission of chronic hypertension or gestational hypertension) with COVID-19 was statistically increased 4.3, aOR (95%CI 1.5–12.4) compared to non-hypertensive women with COVID-19; indicating that COVID-19 in the presence of a hypertensive pregnancy increased the risk of PreE. When adjusted among controls there was no statistical increase in the incidence of PreE between hypertensive and non-hypertensive women, 2.6, aOR (95%CI 0.97–6.8). Similar results were seen among diabetic women with COVID-19, as they had a statistically increased risk of PreE compared to non-diabetic COVID-19 women, 3.9, aOR (95%CI 1.2–12.5). Among controls, those with diabetes did not have a significantly increased risk of developing PreE relative to women without diabetes, 1.3, aOR (95%CI 0.4–4). These results suggest that in the presence of hypertension or diabetes a pregnant woman with COVID-19 is at an increased risk for developing PreE.

There was no statistical difference in infant birthweight between women with COVID-19 and those without (*p* = 0.44, Table 2). Despite this, babies born to women with COVID-19 were more likely to be admitted to the NICU (*p* < 0.0001, Table 2); however, they were not more likely to have a longer NICU stay (*p* = 0.006) compared to women without COVID-19. Complications among infants born to women with COVID-19 were statistically increased regardless of the gestational age at delivery, 7 aOR (95%CI 3.3–15.2). While infants born to women with COVID-19 were not more prone to intraventricular hemorrhage (*p* = 0.67), there was an increase in the number of infants born to women with COVID-19 who had respiratory distress syndrome (22 vs. 9, *p* = 0.006), jaundice (28 vs. 13, *p* = 0.004) or another diagnosis such as hypoglycemia (20 vs. 7, *p* = 0.004).

## 4. Discussion

This study examined the risk factors between COVID-19 and poor maternal and neonatal outcomes in an at-risk population. We demonstrated that in women with chronic hypertension and/or diabetes, COVID-19 infection increases the incidence of PreE. We were also able to demonstrate a relationship between COVID-19 severity and the incidence of PreE. A significant finding in our study is the relationship of maternal obesity, COVID-19 infection and the risk for PreE. As the implications and long-term healthcare concerns for both the mother and newborn when pregnancy is complicated by PreE are not inconsequential, these findings are significant.

Similar to what has been reported by others, in our study women with COVID-19 infection were more susceptible to preterm delivery [11,12]. A recent cohort age-matched study from women delivering in New York with and without COVID-19 reported results similar to ours in that there were no significant differences in the mode of delivery due to COVID-19 [13]. Within that study, there was also a higher NICU admission, increased neonatal complications among babies born to women with COVID-19 relative to women without COVID-19. When women with COVID-19 infection were evaluated by themselves, there was a significant relationship between severity of COVID-19 and development of PreE. This suggests that the more severe the disease state the higher the risk for PreE. As expected, there was a relationship between women who were Black, hypertensive/diabetic and had a high BMI and PreE. However, when women with COVID-19 were matched by comorbidity with women without COVID-19 there was no difference in the incidence of PreE. It was only when the incidence of PreE among hypertensive vs. non-hypertensive women with COVID-19 or diabetic vs. non-diabetic women with COVID-19 were compared that we were able to determine the risk of PreE. The fact that there was no difference in the incidence of PreE among women without COVID-19, regardless of the comorbidity, leads us to believe that for COVID-19 positive pregnant women who have pre-existing diabetes or chronic hypertension they are at an increased risk of developing PreE.

There are several reasons for differences in our study results versus what have been reported by others; one of which is the study population itself. In addition, to having a high rate of preterm birth prior to the COVID-19 pandemic, Mississippi also has a high population of Black women per capita and is constantly ranked as one of the most obese, hypertensive and diabetic states. These factors compound the potential risk and severity of not only negative birth outcomes and risk for PreE but also complications from COVID-19 [8]. Among studies evaluating outcomes similar to those in our study, the percent of women with BMI >30 kg/m^2^ ranges from 7.2–20% whereas our average BMI is >34 kg/m^2^ [14,15]. Similar differences are found when racial groups and hypertensive status is evaluated. Our COVID-19 population consisted of 92% women who identified as Black or Hispanic and 24% of women had chronic hypertension. This is in comparison to studies reporting 4–66.7% Black/Hispanic women [14,15] and 1–5% of these women had chronic hypertension [14,15,16].

The results from this study point to several differences in severity of COVID-19 during pregnancy among our population that has not previously been reported. For instance, women in the current study with COVID-19 infection who were admitted to the ICU were younger than what has previously been reported [17], which speaks to the need for additional investigation with a larger study group to determine why young mothers without medical comorbidities are developing severe illness necessitating ICU admission. Following implementation of universal testing, 51% of our cases were asymptomatic, which is significantly lower than other published reports [18]. Additionally, the previously reported link between COVID-19, severity and Black race, obesity and/or hypertension [19], was not supported in the current study. It could be that like what is seen in areas with negative health outcomes, that in our population of women there’s an increase in susceptibility to COVID-19, however more studies need to be conducted to explore this relationship.

Our study has several strengths and weaknesses. Our first delivery with confirmed SARS-CoV-2 infection was on 14 April 2020. Prior to 13 May 2020, pregnant patients were only tested if they had symptoms of SARS-CoV-2 infection, had a recent travel to a high-risk area, or had a high-risk exposure. On 13 May 2020, we implemented universal COVID-19 testing for all pregnant patients admitted to Labor and Delivery. As such it is possible that some women in our control population may have been asymptomatic for COVID-19. However, when women were matched, all charts were carefully evaluated to ensure that there were no documented notes to indicate that there may have been what we would now consider a suspicion of COVID-19. We also had a small sample size, 100 cases and 100 controls. However, the study was well powered (99%) to address our primary objective.

## 5. Conclusions

To conclude, patients with COVID-19 and chronic hypertension or diabetes mellitus are more likely to develop PreE. Furthermore, the more severe the disease state the higher the risk of PreE. Given the already well established long-term cardiovascular risk factors associated with PreE and the burgeoning factors of long COVID it is important that women affected by COVID-19 are monitored and followed. It is also important to examine the effects that COVID-19 infection during conception/early pregnancy has on placental trophoblast invasion. All of these factors together highlight the importance of early establishment of prenatal care, routine follow up and close monitoring for the development of hypertensive disorders of pregnancy.

## Figures and Tables

**Table 1:** Patient characteristics based on severity of COVID-19.

	Asymptomatic (*n* = 50)	Mild Illness (*n* = 28)	Moderate Illness (*n* = 7)	Severe Illness (*n* = 10)	Critical Illness (*n* = 3)	*p* Value
BMI (kg/m^2^)	32.25 ± 7.7	36.35 ± 10.7	36.77 ± 4.5	34.46 ± 8	47.33 ± 10.5	0.03
Race/Ethnicity (*n*) Black White Hispanic Other	22 4 24 0	16 2 9 1	4 0 2 1	7 0 2 1	2 0 0 1	0.12
HTN/Diabetes (*n*) No Yes	34 16	14 14	3 4	6 4	1 2	0.35
Preeclampsia (*n*) No Yes	44 6	22 6	3 4	7 5	1 2	0.009
Preterm delivery (*n*) No Yes	40 6	23 6	6 4	3 5	1 2	0.006
Trimester at DX (*n*) Second Third	4 46	11 17	0 7	2 8	0 3	0.009
Gest. age Deliv. (wks)	37.53 ± 3	37.78 ± 1.8	38.03 ± 1.2	33.75 ± 4.9	35.1 ± 1	0.02
Preg. Complications (*n*) None PPROM PP Hemor. Shoulder dystocia AKI Other Combination	40 2 3 2 0 1 2	19 0 5 0 0 3 1	5 0 0 0 0 2 0	7 0 1 0 1 0 1	3 0 0 0 0 0 0	0.35
Fetal weight (grams)	3037 ± 753	3180 ± 599	3004 ± 516	1992 ± 1153	2833 ± 504	0.04
Fetal Death (*n*) No Yes	46 4	27 1	7 0	9 1	2 1	0.09
NICU Admission (*n*) No Yes	28 21	17 10	4 3	2 7	1 2	0.13
NICU LOS (days)	11.85 ± 20.9	7.36 ± 8.5	3 ± 0.0	11.86 ± 7.9	6.5 ± 3.5	0.14

HTN = hypertension, DX = Diagnosis, PPROM = preterm premature rupture of membranes; AKI = acute kidney injury; NICU = neonatal intensive care unit; PP = postpartum; LOS = length of stay.

**Table 2:** Characteristics of pregnant women with and without COVID-19.

	Case	Control	*p* Value
Maternal Age (yr)	26.65 ± 6.4	26.81 ± 6.6	0.86
Maternal Race/Ethnicity (n) Black White Hispanic Other	52 6 37 5	54 6 37 5	0.94
BMI (kg/m^2^)	34.25 ± 9.2	34.33 ± 8.9	0.95
Chronic HTN (n) No Yes	76 24	73 27	0.63
Diabetes (n) No Yes	83 17	82 18	0.85
Gest Age Deliv (weeks)	36.82 ± 3.7	37.14 ± 3.1	0.51
Preterm Deliv (weeks) No Yes	75 25	69 31	0.43
Mode of delivery Cesarean Section Vaginal	44 56	40 60	0.57
Preeclampsia No Yes	76 24	74 26	0.74
Pregnancy Complications (n) None PPROM PP Hemor. Shoulder dystocia AKI Other Combination	76 2 9 2 1 6 4	74 3 2 3 1 10 7	0.31
Birthweight (g)	2965 ± 797	2875 ± 837	0.44
Fetal Death No Yes	93 7	98 2	0.17
NICU admission No Yes	52 44	79 18	<.0001
NICU LOS (days)	10.2 ± 15.3	30.7 ± 26.8	0.006

HTN = hypertension, PPROM = preterm premature rupture of membranes; AKI = acute kidney injury; PP = postpartum; LOS = length of stay.

## Data Availability

Data is contained within the article or Supplementary Material.

## Supplementary Materials

The following supporting information can be downloaded at: https://www.mdpi.com/article/10.3390/ijerph192416631/s1, Table S1: Characteristics of COVID-19 positive women admitted to the intensive care unit (ICU); Table S2: Key maternal characteristics of women with COVID-19 and descriptions surrounding infant death.
