# Supplementary material for: COVID-19 Not Hypertension or Diabetes Increases the Risk of Preeclampsia among a High-Risk Population
Source: Int J Environ Res Public Health. 2022 Dec 10;19(24):16631. doi: 10.3390/ijerph192416631 (PMC9779111; doi:10.3390/ijerph192416631)
Supplement: Supplementary file 1 [file ijerph-19-16631-s001.zip › ijerph-1991358-supplementary.pdf]

Table S1. Characteristics of COVID-19 positive women admitted to the intensive care unit (ICU).

	**ICU Admission (n=9)**	**Non-ICU Admission (n=91)**	***p* Value**
**Maternal age (years)**	27.1 ± 6.6	26.78 ± 6.6	0.89
**BMI (kg/m^2)**	39.76± 10.5	33.79± 8.6	0.05
**Race/Ethnicity (n)**			0.03
**Black**	6	46	
**Non-Hispanic White**	0	6	
**Hispanic White**	1	36	
**Other**	2	3	
**Hypertension/Diabetes (n)**			0.48
**No**	4	55	
**Yes**	5	36	
**Gestational age at diagnosis (weeks)**	31.7 ± 3.3	33.97 ± 5.87	0.26
**Gestational age at delivery (weeks)**	34.58±4.32	37.39±2.84	0.008
**Preterm delivery (%)**			0.22
**No**	5	70	
**Yes**	4	21	
**Infant birthweight (g)**	2295.33± 1136.52	3030.88 ± 731.38	0.008
**Infant death**			0.12
**No**	7	86	
**Yes**	2	5	
**NICU Admission (n)**			0.24
**No**	2	50	
**Yes**	5	39	

Table S2. Key maternal characteristics of women with COVID-19 and descriptions surrounding infant death.

**BMI (kg/m2)**	**Race**	**PreE**	**HTN/Diabetes**	**Classification**	**Description**
58.2	Black	Yes	Yes	Critically Ill	Preterm birth (33.3 weeks), maternal diabetic ketoacidosis
42.4	Black	Yes	Yes	Severe	Preterm birth (25.0 weeks)
23.7	Black	No	Yes	Mild	Term birth (37.6 weeks), prenatal diagnosis of severe left sided congenital diaphragmatic hernia
33.7	Black	Yes	Yes	Asymptomatic	Preterm birth (26.3 weeks), maternal diabetic ketoacidosis
27.4	White	No	Yes	Asymptomatic	Term birth (40.1 weeks), prenatal diagnosis of severe left sided congenital diaphragmatic hernia
31	Black	No	No	Asymptomatic	Term birth (37.3 weeks), mother with disseminated intravascular coagulation and category three fetal heart rate tracing. Emergent cesarean delivery for terminal bradycardia with Apgars 0/0/0/2/2.
23.5	Hispanic	No	No	Asymptomatic	Preterm birth (33.4 weeks)
